# Internal Use of Chloroform

**Published:** 1867-12

**Authors:** 


Internal Use of Chloroform.—Dr. Bogue described before the Chicago Medical Society (*Chicago Medical Journal*), the effect of a teaspoonful dose of chloroform, administered in sweetened water to a strong Irishman, for severe colic pain in the abdomen, after five $\frac{1}{8}$-grain doses of morphia had failed to give permanent relief.

Immediately after taking the chloroform, the patient suffered a severe pain in the stomach for half a minute, when he commenced panting violently, laughing, and talking wildly. He then lay upon the bed, continuing to laugh and talk about three minutes; at the expiration of five minutes more, he was fully anæsthetized. For about fifteen minutes, his breathing was slow and stertorous; pulse descending from eighty to forty-eight beats per minute; the veins turgid, lips and face purple.

Sinapisms were applied to the abdomen, and heat to the feet. The pulse and respiration became quite normal in a few moments. Slight vomiting occurred, when the patient slept quietly for nearly an hour and a half. On awaking, he remained entirely free from pain.

Other members gave very favorable reports regarding the effect of chloroform internally administered in cases of nausea and pain in the abdomen.

